# The Role of Preimplantation Genetic Testing for Monogenic Disorders (PGT-M) in Hemoglobinopathy Management—Techniques, Accuracy, and the Balancing of Benefits and Drawbacks

**DOI:** 10.3390/biom15101472

**Published:** 2025-10-17

**Authors:** Rasrawee Chantrasiri, Tawiwan Pantasri, Siriporn Chattipakorn, Nipon Chattipakorn, Sirinart Kumfu, Wirawit Piyamongkol

**Affiliations:** 1 Division of Reproductive Medicine, Department of Obstetrics and Gynaecology, Faculty of Medicine, Chiang Mai University, Chiang Mai 50200, Thailand; rasrawee.c@cmu.ac.th (R.C.); tawiwan.p@cmu.ac.th (T.P.); 2 Cardiac Electrophysiology Research and Training Center, Faculty of Medicine, Chiang Mai University, Chiang Mai 50200, Thailand; siriporn.c@cmu.ac.th (S.C.);; 3 Center of Excellence in Cardiac Electrophysiology Research, Chiang Mai University, Chiang Mai 50200, Thailand; 4 Department of Oral Biology and Diagnostic Sciences, Faculty of Dentistry, Chiang Mai University, Chiang Mai 50200, Thailand; 5 Cardiac Electrophysiology Unit, Department of Physiology, Faculty of Medicine, Chiang Mai University, Chiang Mai 50200, Thailand; 6 The Academy of Science, The Royal Society of Thailand, Bangkok 10400, Thailand; 7 Department of Obstetrics and Gynaecology, Faculty of Medicine, Chiang Mai University, Chiang Mai 50200, Thailand

**Keywords:** preimplantation genetic testing for monogenic disorders, hemoglobinopathies, thalassemia

## Abstract

Preimplantation genetic testing for monogenic disorders (PGT-M) is a powerful tool for identifying genetic disorders prior to gestation. For hemoglobinopathies like thalassemias and sickle cell disease, PGT-M offers a preventative strategy to ensure that only embryos deemed genetically healthy are transferred. A comprehensive review of 22 original articles explores and summarizes the existing evidence on PGT-M techniques in hemoglobinopathies. The review focuses on key aspects such as accuracy, benefits, and drawbacks related to various hemoglobinopathies. Given the limited quantity of DNA obtained from an embryo biopsy, whole genome amplification (WGA) is a critical step for amplifying the sample. One of the available methods of WGA, multiple displacement amplification (MDA) is one of the most widely adopted method with acceptable allele drop-out (ADO) rates for hemoglobinopathies compared with traditional methods. Dealing with ADO constitutes a primary technical obstacle in PGT-M. The failure to amplify one allele in single-cell analysis is a major factor limiting the overall diagnostic accuracy of the procedure. To mitigate this issue, PCR-based and next-generation sequencing (NGS)-based approaches are employed. These methods incorporate linkage analysis with genetic markers such as short tandem repeats (STRs) or single-nucleotide polymorphisms (SNPs) to reduce the risk of incorrect interpretations from ADO and enhance the proportion of conclusive results. A future direction for PGT-M that involves the development of non-invasive methods (niPGT) will be included and discussed.

## 1. Introduction

Hemoglobinopathies are the most common group of monogenic diseases inherited through autosomal recessive transmission [1]. Thalassemia is caused by the absent or reduced production of α or β globin chains. While sickle cell disease is caused by a separate mutation that leads to a structural abnormality in the β-globin chain of hemoglobin [2,3]. Approximately 7% of the global population is estimated to be carriers of hemoglobinopathies [1,4]. Based on the mode of inheritance, there is a 25% probability that the offspring of two carriers will be affected by thalassemia or sickle cell disease. The known prevalence is high in a significant number of areas globally, notably in southeast Asia, the Middle East, the Indian subcontinent, and the Mediterranean area [4]. However, the global distribution of β-thalassemia has been altered by migration, leading to an increased prevalence in traditionally non-endemic populations, such as those in western Europe and North America [5]. The carriers are typically asymptomatic, although some may experience mild clinical manifestations under specific environmental or physiological stress. The severe forms of these diseases place an immense burden on both affected individuals and healthcare systems. Children with severe thalassemia often require lifelong management with blood transfusions and iron chelation therapy [5]. This treatment is critical for managing iron overload, a common and dangerous complication. Without proper management, this can lead to serious health issues, including compromised growth, delayed sexual maturation, endocrine dysfunction, and damage to vital organs like the heart, liver, kidneys, and bones [5,6]. These treatments are expensive and labor-intensive. Furthermore, the mortality statistics highlight the severity of the problem: hemoglobinopathies, including thalassemia, contribute to 3.4% of global deaths in children under the age of five and an even higher 6.4% in Africa [5,7]. This significant social and economic burden underscores the urgent need for effective prevention strategies.

Preimplantation genetic testing for monogenic disorders (PGT-M) has emerged as a promising and impactful solution for preventing the transmission of inherited single-gene disorders. This technique involves the genetic analysis of embryos generated through in vitro fertilization (IVF) or intracytoplasmic sperm injection (ICSI), allowing for the identification and selection of embryos free from a specific genetic mutation. PGT-M offers a definitive method for eliminating the risk of having a child affected by severe conditions like thalassemia or sickle cell disease, thus providing a crucial preventative measure against these debilitating monogenic disorders. This process can provide parents with profound reassurance, as only unaffected embryos are transferred, potentially leading to healthy pregnancies and births. Moreover, PGT-M can facilitate the selection of an unaffected embryo that is HLA-compatible with a previously affected child from a carrier couple. The hematopoietic stem cells derived from the delivered child can then be utilized to treat the affected sibling [8,9].

A significant challenge of PGT-M lies in the extremely limited quantity of input DNA available for analysis, thereby necessitating the application of highly sensitive DNA amplification techniques. The limitations of low DNA input quantities directly correlate with an elevated susceptibility to several technical complications. These include the increased risk of DNA amplification failure, DNA contamination, and allele drop-out (ADO) [9,10]. ADO refers specifically to the non-amplification or preferential amplification of one of the two alleles present in a heterozygous sample, despite the successful amplification of the other. Preferential amplification results in the non-detection of the underrepresented allele, with the detection limit being highly dependent on the analysis platform used [11]. One of the significant concerns is the issue of embryo mosaicism. The low DNA input obtained from the random cell biopsy may be affected by this mosaicism, which can subsequently lead to misdiagnosis and a reduction in the accuracy rate of the PGT-M results [12]. Consequently, the manifestation of any of these technical issues can significantly compromise the integrity and reliability of the diagnostic outcomes. To minimize the occurrence of these issues or enhance their detection, rigorous precautions must be implemented during both the assay setup and its clinical implementation, encompassing the amplification and genetic testing phases. This review aims to summarize and discuss the current evidence focusing on the role of PGT-M techniques, including accuracy, advantages, and disadvantages in various hemoglobinopathies, such as α-thalassemia, β-thalassemia, and sickle cell disease to improve outcomes in conventional PGT-M, as well as less invasive and non-invasive PGT-M.

This comprehensive review was performed using the PubMed database, encompassing publications from January 1990 to May 2024. This search was undertaken utilizing specific search terms such as “Preimplantation genetic testing for monogenic disorders*”, “Thalassemia*”, and “Hemoglobinopathies*. The search yielded a total of 22 relevant original articles, all of which were subsequently incorporated into this review. During the preparation of the manuscript, the authors used Gemini version 2.5 Flash for grammar, structure, and English proofreading.

## 2. Overview of Hemoglobinopathies

α-Thalassemia is caused by deletions or single nucleotide substitution in the *HBA2* and *HBA1* genes located at 16p13.3, leading to insufficient synthesis of the α-globin chains [13]. The most severe manifestation of this disorder is Hb Bart’s disease, also known as Hb Bart’s hydrops fetalis syndrome. This syndrome arises from the complete absence of α-globin chain synthesis due to homozygosity for α^0^-thalassemia deletion. The condition is lethal, causing intrauterine fetal death and posing significant risks to the mother, including eclampsia, shoulder dystocia, and postpartum hemorrhage [14]. Hb H disease represents a severe form of α-thalassemia that is compatible with postnatal survival. This condition is caused by the inheritance of an α^0^-thalassemia deletion on one copy of chromosome 16 and an α^+^-thalassemia deletion on the homologous chromosome [4,15]. β-thalassemia is an inherited hematological disorder resulting from single nucleotide substitutions or deletions within the β-globin genelocated on chromosome 11p15.4 [16,17]. These mutations lead to the deficient ( β^+^) or absent ( β^0^) synthesis of β-globin subunits. Clinically, heterozygous β-thalassemia typically presents with mild or asymptomatic anemia, whereas homozygous β-thalassemia manifests as severe, early-onset anemia requiring lifelong blood transfusions [18]. One of the most prevalent subtypes of severe thalassemia worldwide is -β-thalassemia/Hb E disease, which accounts for almost 50% of all cases. Hb E is attributed to a single nucleotide substitution, a change from GAG to AAG at codon 26 of the *HBB* gene. Patients with β-thalassemia/Hb E disease experience severe anemia shortly after birth, which also necessitates frequent blood transfusions. Conversely, sickle cell disease is caused by a separate mutation that leads to a structural abnormality in the β-globin chain of hemoglobin [2,3]. This abnormal hemoglobin causes red blood cells to become rigid and adhesive, resulting in a range of health complications. Individuals with sickle cell disease often present with chronic pain, fatigue, anemia, and heightened susceptibility to infections [19].

## 3. Preimplantation Genetic Testing and Stages of Embryo Biopsy

The history of preimplantation genetic testing and embryo biopsy is a revolution spanning three decades, transitioning from initial cleavage stage techniques to the current gold standard of trophectoderm biopsy at the blastocyst stage. First, in the 1960s, embryo biopsy has its origin to sex the embryos of the farm animals for breeding. Later, in 1968, Gardner and Edward performed a trophectoderm biopsy in a rabbit and developed the concept of sex selection in X-linked inherited disease which avoid transferring the male embryos. In late 1989 to 1990, the first PGT was successfully implemented by Handyside et al. for embryo sex selection to avoid X-linked inherited disorders by blastomere biopsy [20], while in 1990, Verlinsky et al. [21] were the pioneers of the first polar biopsy for PGT for alpha-1-antitrypsin deficiency using PCR techniques and simultaneously with Dokras et al. [22] proposed the feasibility of trophectoderm biopsy. Polar body biopsy was gradually discontinued because its utility was restricted to providing information solely on the maternal chromosomal component. This shift favored blastomere and trophectoderm biopsy due to the absence of well-established supporting data for polar body biopsy, as well as its inherent possibility of diagnostic inaccuracy and failure [23,24,25]. Subsequent application of extended embryo culture protocols, facilitated by the development of more complex media or new generation sequential media, has significantly enhanced the efficiency and reported high rates of development to the blastocyst stage. Comparative studies have demonstrated the clinical superiority of later-stage biopsy. One study compared pregnancy outcomes between embryos that underwent cleavage-stage blastomere biopsy and those that were not biopsied. This research indicated that blastomere biopsy may lead to a significant reduction in implantation potential, whereas trophectoderm biopsy demonstrated no such significant negative impact on implantation [26]. Moreover, blastocyst biopsy, which involves the removal of multiple trophectoderm cells rather than a single blastomere, resulted in a reduced rate of ADO [25].

Furthermore, trophectoderm biopsy offers distinct advantages regarding clinical resource allocation. This approach is considered relatively more cost-effective and less labor-intensive because the biopsy is selectively performed only on those embryos that have demonstrated the developmental competence to reach the blastocyst stage. The current practice of trophectoderm biopsy typically involves the retrieval of five to ten cells, which generally provides ample material for comprehensive genetic analysis. PGT is broadly categorized into three main types: PGT for monogenic diseases (PGT-M), PGT for aneuploidy (PGT-A), and PGT for structural chromosomal rearrangements (PGT-SR). PGT-M focuses on identifying specific genes associated with particular monogenic disorders through embryo analysis. PGT-A is utilized to detect chromosomal aneuploidy, which can lead to implantation failure or pregnancy loss. Finally, PGT-SR is employed to identify unbalanced structural rearrangements, such as reciprocal and Robertsonian translocations. While each type serves distinct purposes, they possess overlapping aims, ultimately seeking to improve the likelihood of a successful pregnancy [9,27,28]. Couples undergoing PGT are required to undergo assisted reproductive technologies, a process comprising several key stages. This typically begins with genetic counseling, followed by ovarian stimulation and oocyte retrieval. Fertilization is often performed via intracytoplasmic sperm injection (ICSI), potentially reducing the risk of contamination from cumulus cells and sperm. The process then proceeds to embryo culture and subsequent embryo biopsy [1,9].

## 4. The Technical Outcomes of PGT-M for Hemoglobinopathies

After obtaining biopsied cells, genetic analysis typically involves several key steps. First, cell lysis is performed to extract DNA from the cellular membranes. This is followed by whole genome amplification (WGA), which is crucial for amplifying the minute starting sample to create millions of copies of the entire genome. Alternatively, targeted amplification can be performed, focusing only on specific genes of interest. Given the need for a highly specific and efficient amplification method to yield sufficient targets for accurate diagnosis, WGA is the more common technique used in PGT-M [29]. Consequently, all recruited studies in this context utilized WGA techniques to increase DNA sample quantities. Finally, various methods are employed for molecular genetic analysis, enabling a comprehensive examination of the amplified DNA. A summary of WGA use for PGT-M is shown in Table 1.

### 4.1. Whole Genome Amplification for PGT-M

WGA provides sufficient DNA templates from minute DNA samples obtained from embryonic cell biopsies, enabling subsequent amplification procedures or other downstream applications [9]. WGA is primarily achieved through two main methodological approaches: polymerase chain reaction (PCR)-based reactions and the multiple displacement amplification (MDA). Common PCR-based techniques include degenerate oligonucleotide primed PCR (DOP-PCR), PicoPlex, and multiple annealing and looping-based amplification cycles (MALBAC).

PCR approaches achieve whole-genome amplification through a non-selective amplification process. The technique involves multiple cycles with variable annealing temperatures to increase the likelihood that primers bind to a broad range of genomic sequences, aiming for even and proportional whole-genome amplification. Despite the exponential amplification, PCR approaches often result in low gene coverage [30]. This is because the rapid, exponential amplification can lead to poor uniformity, which negatively impacts overall coverage diagnosis [29,31]. MDA is the most common isothermal whole-genome amplification for PGT. This technique utilizes random primers that are resistant to exonuclease activity, with amplification proceeding via strand displacement synthesis mediated by φ29 DNA polymerase. The φ29 DNA polymerase, possessing potent proofreading activity, ensures high fidelity of the amplification products and consistent yield across diverse templates. This technique offers several advantages, including ease of operation, minimized human error, effective prevention of DNA sample loss, amplification of long DNA fragments, reduced waste of DNA samples, and broad commercial availability [29,32,33,34]. Even though MDA is currently the recommended method for PGT-M [9], other techniques remain in use at many centers depending on individual center’s specific capabilities and preferences. The various technical and clinical outcomes of PGT-M based on PCR are shown in Table 2. Studies on PGT-M for β-thalassemia and sickle cell disease using PCR have reported varying technical outcomes. Specifically, amplification rates ranged from 67.3% to 95.7% in studies using blastomere biopsy and from 86% to 100% in studies using trophectoderm biopsy. ADO rates also differed by biopsy method, with blastomere biopsy studies reporting rates between 0.01% and 19%, while trophectoderm biopsy studies showed lower rates of 0% to 2.7% [8,35,36,37,38]. MDA can serve as an effective initial step for WGA to improve diagnostic efficacy by increasing the amplification rate and reducing the ADO rate. According to one large study comparing PCR and MDA techniques for β-thalassemia, no significant difference in amplification rate was observed between the two methods [39]. However, the MDA technique demonstrated greater diagnostic accuracy and a lower ADO rate than PCR [39], potentially because of its reduced genome target bias. Building on these advantages and working with scarce DNA quantities, employing MDA as a preliminary step can improve diagnostic efficiency by 8.84% for PGT-M for β-thalassemia [39].

The α-globin gene lies within a 135–155 kb GC-rich region [40], which makes it refractory to amplification compared to other single gene defects. This presents a significant challenge for PGT-M in α-thalassemia. MALBAC and other PCR approaches (particularly PicoPlex) have identical mechanisms. The paper on MALBAC only advanced the understanding of stem loop suppression, which is an intrinsic mechanism in both DOP and PicoPlex. One study comparing MDA and MALBAC for deletional α-thalassemia found MDA was superior to MALBAC for PGT-M due to a higher consistency rate [13], although other studies [10,13,41,42] found no statistically significant difference in either amplification success rate or ADO rate between these two methods [13].

In this review, it was found that MDA is a more effective and accurate technique for WGA in the context of PGT for hemoglobinopathies, compared with MALBAC and PCR. Specifically, MDA demonstrates a greater consistency rate.

### 4.2. Genetic Testing Techniques

PGT for a specific gene-related condition is typically performed using two primary approaches: a direct method, which involves the specific detection of the causative gene defect and an indirect method, which utilizes haplotype-based linkage analysis. This indirect method tracks the inheritance of polymorphic genetic markers such as short tandem repeats (STRs) or single nucleotide polymorphisms (SNPs) that are closely situated to the disease-causing gene, allowing for the construction of a unique family haplotype that consistently co-segregates with the pathogenic allele [27]. Crucially, the indirect method is employed to confirm the direct test result, significantly mitigating the risk of misdiagnosis due to ADO during single-cell amplification. Therefore, current techniques and technological advancements aim to reduce the ADO rate while simultaneously increasing diagnostic accuracy. More recently, next-generation sequencing is considered among the most effective technologies currently employed for performing PGT-M [9]. However, there remains a role for other genetic testing methods, contingent upon patients’ economic considerations and the center’s specific capabilities. A summary of methods utilized for genetic testing in PGT-M is shown in Table 2 and Table 3.

#### 4.2.1. PCR-Based Method (Table 2)

In a 1999 study, denaturing gradient gel electrophoresis (DGGE) was employed for PGT-M. DGGE is considered advantageous for mutation detection as it facilitates the simultaneous analysis of multiple mutations within a single PCR fragment, thus enabling the characterization of compound genotypes. However, this method yielded only a moderate conclusive result rate of 50% for β-thalassemia, coupled with a high ADO rate of 19% [38]. Consequently, DGGE is not considered an optimal genetic testing approach for PGT-M applications [38]. The application of multiplex PCR for the detection of Hb H disease, beta-thalassemia, and sickle cell disease proved highly effective [4,43]. This methodology consistently produced conclusive outcomes with rates spanning 90.8% to 100% [4,43].

**Table 1:** Comparative outcomes of whole genome amplification (WGA) for preimplantation genetic testing for monogenic disorders (PGT-M).

Disease (*n* of Embryos)	Mutations	Methods	Results		PGT				Pregnancy Outcomes				Interpretation	Ref.
			Amplification Rate (%)	ADO Rate (%)	Conclusive (%)	Unaffected (%)	Euploid (%)	HLA Matched (%)	Number of CP/FET (%)	Number of LB/ FET (%)	PGT Accuracy (PND)			
											Thal (%)	Ploidy (%)		
Comparative Outcomes for Whole Genome Amplification														
Blastomere Biopsy														
β thalassemia (4) * *blastomere biopsy*	IVSII-1 (G→A) CD 39 (C→T) • STR for HBB D11S988, D11S4181, D11S2362, D11S4891, D11S1760, D11S1338 • STR for HLA MOG, D6S510, MIB, LH1, HLA BC, DRA CA, RING3, D6S1560	• PCR Compare lysis method ① Alkaline lysis buffer method ② Lysis buffer contained NaOH ③ *N*-lauroylsarcosine salt buffer method ④ Proteinase K/SDS lysis method • Multiplex fluorescent PCR • PGT-M	① 96.5 ② 96.5 ③ 84.4 ④ 98.2	① 7.1 ② 8.9 ③ 17.3 ④ 1.7	4/4 (100)	3/4 (75)	N/A	2/4 (50)	1/1 (100)	1/1 (100)	N/A	N/A	④ could be used as a more acceptable lysis method with a lower rate of ADO and less amplification failure rates.	[35]
**Trophectoderm Biopsy**														
β thalassemia (2315) * Trophectoderm biopsy	41–42 (–CTTT), −28(A→G), CD 17 (A→T), IVS-II-654, CD 71–72 (+A), −29 (A→G), CD 26 (G→A), CD43 (G→C), CD14–15(+G), CD 27–28 (+C), −32(C→A), −30(T→G), IVS-I-1 (G→T), IVS-I-5 (G→C), CAP+40– +43 (–AAAC) and CD 31(–C)	① MDA group (*n* = 1463) • MDA (REPLI-g) • Single plex PCR + reverse dot blot • PGT-M	N/A	↓	1419/ 1463 (96.99)	1054/ 1419 (74.28)	N/A	N/A	123/ 281 (43.7)	102/ 281 (35.9)	N/A	N/A	Using MDA as the 1st step in PGT-M for β-thalassemia could increase diagnostic efficiency.	[39]
		② PCR group (*n* = 852) • PCR • Multiplex nested PCR + reverse dot blot analysis • PGT-M	N/A	↑	751/ 852 (88.15)	488/ 751 (64.98)	N/A		79/ 192 (41.1)	67/192 (34.9)	N/A	N/A		
α thalassemia (253) * Trophectoderm biopsy	--^SEA^, α^CS^, α^3.7^, - α^4.2^	• Compare ① MALBAC vs. ② MDA (REPLI-g) • SNP or gap-PCR/Sanger sequencing • PGT-M/PGT-A	① 71/72 (98.61) ② 179/181 (98.89) (*p* value = 0.85)	① 2.27% ± 3.57% ② 0.97% ± 1.4% (*p* value = 0.45)	Consistency between SNP and gap PCR/sanger sequencing			N/A	24/32 (75) 13/24 ongoing pregnancy	11/32 (34.3)	11/11 (100)	11/11 (100)	MDA was superior to MALBAC for PGT of deletional α-thalassemia in consistency between SNP/PCR and sanger sequencing, but no significant difference in amplification rate and ADO rate.	[13]
					① 50% ② 83.43%									

Abbreviation: ADO, allele drop-out; CGH, comparative genomic hybridization; CP, clinical pregnancy; EB, embryo; ET, embryo transfer; LB, livebirth; MALBAC, multiple annealing and looping-based amplification cycles; MDA, multiple displacement amplification; n, number; N/A, not applicable; NGS, next-generation sequencing; PCR, polymerase chain reaction; PGD, preimplantation genetic diagnosis; PGT, preimplantation genetic testing; PGT-A, preimplantation genetic testing for aneuploidy; PGT-M, preimplantation genetic testing for monogenic disorder; SNP, single nucleotide polymorphism; STR, short tandem repeat; WGA, whole genome amplification; *, biopsy stage.

##### Short Tandem Repeat-Based Linkage Analysis

The limited availability of informative STRs within the target region and its adjacent upstream and downstream areas presents a significant challenge. This limitation can result in insufficient linkage markers and consequently reduces accuracy of haplotype analysis [16]. Conclusive PCR-STR results for α-thalassemia, β-thalassemia, and sickle cell anemia ranged from 84.2% to 100% [8,16,37,43,44], with ADO rates spanning 0.01% to 7.3% [8,37], as shown in Table 2. However, STR-based haplotyping had low detection throughput and a long test work up time [45,46]. While STRs have inherent limitations, PCR-based STR analysis can significantly reduce the misinterpretation from ADO compared to standard PCR alone. Crucially, the inclusion of STRs facilitates linkage analysis, thereby strengthening the interpretative reliability and enabling a successful diagnosis even when a technical ADO event affects the primary gene mutation.

##### Single Nucleotide Polymorphism-Based Haplotyping

Preimplantation haplotype linkage analysis has, in recent times, largely shifted to employing the abundant SNPs found within the human genome as genetic markers. Karyomapping is a powerful technique that performs genome-wide haplotype analysis of the parents using a high density of SNPs. These SNPs are typically selected based on high heterozygosity and regular spacing across the genome, rather than being clustered around a specific gene. Karyomapping operates by tracing the inheritance of large parental haplotype blocks (meiotic blocks) and not the proximity of a single SNP to the causative gene. The significant number of SNPs utilized in this method helps reduce the impact of ADO. Additionally, karyomapping offers the valuable capability of identifying CNV and tracing parental origins [16,45]. When compared to conventional PCR-STR analysis for β-thalassemia, karyomapping demonstrated a higher conclusive result rate of 96.36–100%, whereas conventional PCR-STR ranged from 91.81% to 94.5% [6,16], as shown in Table 2.

Karyomapping, however, also presents certain limitations. For example, as its principle is rooted in SNP-based haplotyping, chromosomal recombination remains a primary contributor to reduced accuracy and potential misdiagnoses, thereby presenting diagnostic challenges. Consequently, conventional STR analysis remains a valuable option in cases where the data from current SNP array designs do not provide sufficient informative markers for reliable linkage analysis, which could otherwise compromise diagnostic interpretation [16]. For PCR-SNP, both the conclusive results and the SNP detection rate can be influenced by the WGA technique employed. One study, which compared MDA and MALBAC prior to PCR-SNP, reported higher SNP detection rates and more conclusive results in the MDA group [13] Even though STRs have been reported to have low detection throughput and long test workup time, it is not always justified to consider SNP-based haplotyping methods as a superior alternative [45,46]. The choice of haplotyping linkage analysis depends on the specific protocols of each center and the type of genetic testing being performed.

**Table 2:** Various technical and clinical outcomes of PCR-based PGT-M.

Disease (*n* of Embryos)	Mutations	**Methods**	**Results**		**PGT**				**Pregnancy outcomes**				**Interpretation**	**Ref.**
			Amplification Rate **(%)**	**ADO Rate** **(%)**	**Conclusive (%)**	**Unaffected (%)**	**Euploid** **(%)**	**HLA Matched (%)**	**Number of CP/FET** **(%)**	**Number of LB/** **FET** **(%)**	**PGT Accuracy** **(PND)**			
											**Thal (%)**	**Ploidy** **(%)**		
PCR based method (Directed variant analysis)														
β thalassemia/sickle cell (907) * blastomere biopsy	β mutation: IVSI 110 (G→A)	• WGA • Multiplex PCR • PGT-M	N/A	N/A	824/907 (90.8)	602/824 (73.1)	N/A	164/824 (19.9)	102/331 (30.8)	98/331 (29.6)	98/98 (100)	N/A	Multiplex PCR could achieve high conclusive results.	[43]
β thalassemia (52) * blastomere biopsy	β mutation: Codon 39, IVS-I-1, IVSII-1, IVSI-6, IVSI-110, FSC 6, δβSic	• PCR, nested-PCR • DGGE • PGT-M	35/52 (67.3)	10/52 (19)	26/52 (50)	17/52 (32.69)	N/A	N/A	6/9 (66.7)	2/9 (22.2)	4/4 (100)	N/A	The PGD cycles for thalassemia major was carried out using DGGE for mutation analysis with a moderate conclusive result.	[38]
Hb H disease (37) * Trophectoderm biopsy	--^SEA^*/-* α^3.7^	• MDA • REPLI-g Single Cell Kit (QIAGEN) • Multiplex fluorescent PCR • PGT-M	HBA2 97.3, HBA1 100 Internal control 0	HBA2 2.7, HBA1 0 Internal control0	37/37 (100)	37/37 (100) 15 normal, 22--*/-* α^3.7^	N/A	N/A	N/A	N/A	N/A	N/A	A novel PCR primer ( α^+^ thalassemia −3.7 kb deletion) was invented to detect Hb H disease using multiplex fluorescent PCR with high conclusive results.	[4]
PCR based with linkage analysis														
• PCR—Short tandem repeat (PCR-STR)/HLA matching														
β thalassemia/sickle cell (331, 38) * blastomere biopsy	β- mutation: HBS, Codon 30 (A>G), Codon 6 (–A), IVS-I-110 (G>A) Hb S [β6(A3)Glu-Val] mutation Codon 39 (C>T) IVS-I-1 (G>A), IVS-I-5 (G>A) IVS-I-6 (T>C) IVS-II-745 (C>G) IVS-II-1 (G>A) –44 bp deletion –28 (A>G) –101 (C>T)	• PCR (Multiplex Hotstar taq) • Multiplex PCR with/or without STR Novel primer: D11S4891-① D11SZ2-② D11S2362-③ β gene-④ • PGT-M	① 91 ② 91 ③ 86 ④ 92	① 4.8 ② 0.01 ③ 4.7 ④ 7.3	279/331 (84.2)	with STR 174/279 (62.3)without STR 149/279 (53.4)	N/A	N/A	14/108 (12.9)	N/A	N/A	N/A	STRs marker facilitated robust assignment of β hemoglobinopathy genotypes, increasing the number of transferrable embryos by decreasing ADO rate.	[37]
β thalassemia (278) * blastomere biopsy	β-mutation: IVSI 110 (G>A)	• Multiplex PCR • Mini sequencing-based HLA typing combined with HLA STR haplotyping • PGT-M	266/278 (95.7)	12/278 (4.31)	250/266 (94)	15 with HLA matched	N/A	255/266 (95.9)	N/A	N/A	N/A	N/A	Minisequencing-based HLA typing combined with HLA STR haplotyping was a reliable strategy for preimplantation HLA matching.	[8]
β thalassemia (51) * blastomere biopsy	β-mutation	• WGA • The optimized one-step touchdown multiplex PCR • PGT-M/PGT-A	48/51 (94.11)	N/A	44/51 (86.27)	With HLA matched 5/44 (11.36)	N/A	N/A	1/8	2/8	N/A	N/A	The optimized one-step touchdown multiplex protocol showed good outcomes and significantly reduced time.	[36]
			Timing											
			The optimized one-step touchdown multiplex		7 h									
			HLA-PGD procedure, employing WGA		17 h									
α thalassemia (440, 89) * Trophectoderm biopsy	--^SEA^	• MDA REPLI-g Single Cell Kit (QIAGEN) • STR haplotyping Located on Chr 16:D16S521, 16PTEL03, HBA572 Located within HBA gene: 16PTEL05, 16PTEL06 • PGT-M/PGT-A	424/440 (96.36)	N/A	424/424 (100)	Unaffected and Euploid 278/424 (65.5)		N/A	65/89 (73.0)	36/89 (40.4)	36/36 (100)	36/36 (100)	Using STR as markers for PGD had feasibility, credibility, and high conclusive results.	[44]
PCR-Single nucleotide polymorphisms (PCR-SNP)/karyomapping														
β thalassemia /Hb E disease * Trophectoderm biopsy	2 families ① c.41_42delTCTT & c.26G>A ② c.17 & c.26G>A	• MDA • Multiplex F-PCR and karyomapping • PGT-M/ • PGT-A	13/13 (100)	N/A	13/13 (100)	7/13 (53.8)		10/13 (76.9)	N/A	1/1 (100)	1/1 (100)	1/1 (100)	Multiplex PCR with mini- sequencing confirmed karyomapping results can be performed in β-thalassemia and bthal/hbE disease.	[6]
			11/11 (100)		11/11 (100)	8/11 (7.27)		6/9 (66.6)		1/1 (100)	1/1 (100)	1/1 (100)		
β thalassemia (1205) * Trophectoderm biopsy	β mutation	Karyomapping human karyomapping-12beadschip (illumina) (*n* = 851) • HBB • HLA	22	N/A	820/851 (96.36)	614/820 (74.88)		551/827 (66.6)	N/A	N = 42	N/A	N/A	Karyomapping contributed to accurate selection of matched embryos, along with aneuploidy screening.	[16]
			N/A		820/851 (96.36)	-		-	N/A					
		PCR-STR (*n* = 354) • HBB-RDB • HBB-STR • HBB-HLA	20		333/354 (94.35)	257/334 (76.95)		-	-	N = 12	N/A	N/A		
			N/A		325/354 (91.81)	245/325 (75.38)		-	-					
			N/A		326/354 (92.09)	-		-	48/334 (14.37)					

Abbreviation: ADO, allele drop-out; CGH, comparative genomic hybridization; CP, clinical pregnancy; DGGE, denaturing gradient gel electrophoresis; EB, embryo; ET, embryo transfer; HLRS, haplotyping by linked read sequencing; LB, livebirth; MALBAC, multiple annealing and looping-based amplification cycles; MDA, multiple displacement amplification; *n*, number; N/A, not applicable; NGS, next-generation sequencing; PCR, polymerase chain reaction; PGD, preimplantation genetic diagnosis; PGT, preimplantation genetic testing; PGT-A, preimplantation genetic testing for aneuploidy; PGT-M, preimplantation genetic testing for monogenic disorder; SNP, single nucleotide polymorphism; STR, short tandem repeat; WGA, whole genome amplification; *, Biopsy stage.

##### HLA Matching

Currently, survival for patients with β-thalassemia major largely depends on receiving hematopoietic stem cell transplantation (HSCT). Unfortunately, finding a bone marrow donor with compatible human leukocyte antigen (HLA) typing is often both challenging and time-consuming. This difficulty stems from the highly polymorphic nature of HLA genes, which are located on the short arm of chromosome 6 [47]. PGT offers a crucial solution. It not only allows for screening for thalassemia-unaffected embryos but also enables the selection of embryos with an HLA profile compatible with the affected child. This capability empowers families to conceive and deliver ‘savior babies,’ providing a vital source of compatible stem cells for their affected siblings [41]. PGT for HLA typing initially began with minisequencing-based HLA typing combined with HLA-STR haplotyping. This approach, when performed on single blastomeres following WGA, is a reliable strategy, with reported validation success rates typically above 94% and a rate as low as 4.3%[8]. However, this indirect STR method still presented inherent limitations including the limited number of STR systems that can be analyzed in a single reaction. This is further complicated by the high variability of STR markers and their loci across different ethnic groups, which necessitates a time-consuming process for designing individualized markers and specific panels for diverse populations [48,49]. More recently, NGS-based HLA typing has emerged (Table 3). Initial applications, starting with a single blastomere for PGT-HLA following MDA-based WGA, showed promising 100% conclusive results [10,47].

#### 4.2.2. Next-Generation Sequencing-Based Methods (Table 3)

Next-generation sequencing (NGS), also known as massively parallel sequencing, is a powerful technology that has revolutionized genomics. NGS enables rapid and cost-effective sequencing of large numbers of DNA fragments. The feature of high-throughput sequencing is considered a hallmark of NGS, irrespective of the platform utilized [50,51]. Integrating NGS-based method with SNP genotyping provides a highly efficient diagnostic platform capable of yielding comprehensive results from a single biopsy. This multi-purpose method allows for the simultaneous assessment of conditions such as α- and β-thalassemia, HLA matching, and aneuploidy, effectively eliminating the need for repeated biopsy interventions [10,41,52]. Before NGS testing, MDA is considered the most suitable amplification method due to its higher DNA yield. Although MDA-based WGA can introduce significant amplification bias, this defect may be minimized through linkage analysis that incorporates numerous SNPs [41]. Regarding performance metrics, the rate of conclusive results from the NGS-based approach typically falls within the range of 81.48% to 100% [1,9,31,32,33,34,37]. However, the ADO rate observed with NGS testing for alpha-thalassemia, beta-thalassemia, and sickle cell disease has been reported to range from approximately 0% to 4.8% [1,31,33,34,37].

Linkage analysis typically necessitates DNA samples from the prospective parents, the proband, or other relevant family members. This allows for the establishment of informative markers and the identification of the couple’s haplotypes. Subsequently, these informative markers are utilized to analyze the embryos, thereby confirming their haplotypes and determining the presence of familial genetic mutations. However, challenges arise with de novo mutations or when affected familial members are unavailable. In such instances, conventional linkage analysis techniques cannot be performed. Fortunately, haplotyping with linked-read sequencing (HLRS) offers a solution by enabling the construction of carrier haplotypes without requiring samples from additional family members but use of sibling embryos for linkage patterns [45,53]. HLRS comprises two primary processes. The first, a pre-examination phase, involves utilizing genomics technology to perform linked-read sequencing and whole-genome haplotyping. The second, a clinical examination phase, focuses on selecting informative SNPs from parental samples and embryos for determining the embryo’s haplotype. Notably, trophectoderm cells are amplified using PicoPlex for WGA, achieving a 100% amplification rate. This process has shown no detectable ADO and a very high conclusion rate [53].

Recently, another technology, long-read sequencing on the single molecule real-time (SMRT) platform, has become widely available. This method enables SNP haplotype linkage analysis of embryos for couples at risk of transmitting beta-thalassemia without requiring samples from additional family members. The location of the HBB gene on the short arm of chromosome 11, close to the telomere, limits the number of detectable nearby SNPs. Wu et al. used long-fragment PCR on the SMRT platform to amplify regions of approximately 4–7 kb in the HBB gene, linking up consecutive SNPs through this region. Nevertheless, the ADO rate associated with this approach was relatively high, with 10 out of 32 embryos failing haplotype linkage analysis in the core region (31.25%) [45]. However, the SMRT platform was a potential tool for preimplantation haplotype linkage analysis for those who cannot employ conventional NGS-SNP [45].

In conclusion, SNP-NGS stands out as the most promising and widely applicable method for PGT-M for hemoglobinopathies. Its key advantages lie in consistently yielding low ADO rates and high conclusive results. A notable consideration for NGS, however, is its typical requirement for a proband or affected relatives’ samples, with exceptions being the SMRT platform and HLRS, which can proceed without additional family members. Many of these technologies are not universally accessible in all centers and can be cost-prohibitive in certain regions. In such scenarios, alternative approaches using STRs or SNPs in conjunction with fragment analyses or Sanger sequencing offer viable solutions, often delivering comparable results with moderate to high conclusiveness.

## 5. The Future Directions

### Noninvasive to Minimally Invasive Preimplantation Genetic Testing for Monogenic Disorders (Table 4)

While trophectoderm biopsy is generally considered safe, its invasive nature may have some minimal effects on the implantation potential or developmental competence of the resulting embryos [54,55]. Moreover, TE biopsy still presents several significant challenges. These include the laborious and skill-dependent nature of the procedure, its inherent invasiveness, and the potential for harm to the embryo. Furthermore, there is a risk of sampling bias, as the removed cells may not accurately represent the remaining TE cells or the status of the ICM [55,56] in any associated chromosome profiles. These limitations have consequently spurred interest in the development of non-invasive preimplantation genetic testing (niPGT) methods. One promising avenue involves the analysis of cell-free DNA (cfDNA) present in the spent culture medium in which embryos are grown [57]. However, the precise origin of DNA within spent embryo culture medium remains incompletely characterized, and it is unclear whether this DNA is of embryonic origin. One plausible hypothesis is that embryos release DNA into the surrounding culture medium during blastocyst development. Cells from the ICM and TE release DNA into the blastocoel fluid and culture medium via cell lysis, apoptosis, or shedding. Lysis can lead to the release of intact chromosomes and fragmented DNA after nuclear degradation [58]. This non-invasive approach holds the potential to yield genetic information with less necessitating embryonic manipulation, thereby offering a safer and potentially more accessible alternative to conventional methods. Furthermore, niPGT demands less technical expertise and presents a reduced risk to embryonic viability, as trophectoderm biopsy has been associated with potential adverse effects on embryo health and subsequent implantation [15].

Three types of specimens were studied for niPGT-M: blastocoel fluid (BF), spent culture medium (SCM), and SCM containing BF. Based on existing studies, the greatest DNA yields were associated with SCM containing BF, SCM, and BF, respectively. The SCM containing BF was collected following laser lysis of the zona pellucida and subsequent release of the BF into the SCM [56]. The rationale explaining the highest DNA yield from this method is that the laser also damages trophectoderm cells, causing them to lyse. A single study demonstrated that the concentration of DNA in SCM increases over time, with samples collected on Day 6 showing the highest DNA concentration compared to those from Day 4 and Day 5 [59]. However, a comparison of diagnostic efficacy between blastomere biopsy and SCM samples from different collection days yielded a different ranking. The results indicated that diagnostic efficacy was highest for SCM collected on Day 5, followed by SCM from Day 6, and finally SCM from Day 4 compared to the blastomere [59]. The concordance rate compared with TE biopsy is correlated with the DNA concentration: SCM containing BF samples exhibited a 100% concordance rate, SCM samples ranged from 19.67 to 90.16%; and BF samples showed 13.3% (Table 4). Ultimately, niPGT has become a viable alternative to trophectoderm biopsy. While there are limited number of studies in this area, this review suggests that the most appropriate specimens, ranked in descending order of DNA concentration, are those containing both SCM and BF, followed by SCM, and finally BF. Although the WGA technique is believed to be a contributing factor, there is currently no study that directly compares niPGT outcomes using different WGA methods. The future clinical application of niPGT is significantly hindered by the paucity of available DNA in the non-invasive samples. Addressing this fundamental limitation necessitates concentrated efforts in three key areas: optimization of specimen collection methodologies and material types, enhancement of whole genome amplification protocols, and the refinement of downstream genetic testing techniques. Furthermore, the implementation of niPGT-M imposes specific requirements on laboratory culture conditions. While some reports suggest that an individual culture system may not be inferior [60], the current standard of practice in many embryology laboratories involves a group culture approach. For niPGT-M, however, an individualized embryo culture system is required after day 3 of development. A final consideration pertains to the specimen acquisition technique of blastocoel fluid. The blastocoel fluid requires the embryologist to perform blastocentesis using an ICSI needle. To mitigate the critical risk of cross-contamination from exogenous cellular material, a new, sterile ICSI needle must be employed for each individual blastocyst. This operational requirement inherently introduces a significant increase in the consumable costs for the fertility center.

**Table 3:** Various technical and clinical outcomes of PGT-M based on next-generation sequencing (NGS), haplotyping by linked-read sequencing (HLRS) and long-read sequencing technologies.

**Disease (** ***n*** **of Embryos,** ***n*** **of Family)**	**Mutations**	**Methods**	**Results**		**PGT**				**Pregnancy Outcomes**				**Interpretation**	**Ref.**
			**Amplification Rate**	**ADO Rate** **(%)**	**Conclusive (%)**	**Unaffected (%)**	**Euploid** **(%)**	**HLA Matched (%)**	**Number of CP/ET** **(%)**	**Number of LB/** **ET** **(%)**	**PGT Accuracy** **(PND)**			
											**Thal (%)**	Ploidy (%)		
NGS based method														
/β double thalassemia (112, 12)	α mutation: --^SEA^, α^CS^ α, - α^3.7^, - α^4.2^ β mutation: CD 17, CD 41–42, BE, B-28, B IVSII-654	• MDA • NGS based SNP haplotyping • PGT-M/PGT-A	112/112 (100)	N/A	107/112 (95.5)	56/107 (52.3)	Euploid & unaffected 37/56	N/A	11/16 (68.8)	11/16 (68.8)	7/7 (100)	7/7 (100)	NGS-SNP achieved high conclusive results and eliminated the need for multiple biopsies.	[52]
**α/β** double thalassemia (35, 3)	α mutation: --^SEA^, - α^3.7^, - α^4.2^ β mutation: CD 17, CD 41–42	• MDA REPLI-g Single Cell Kit (QIAGEN) • NGS based SNP haplotyping • PGT-M/PGT-A with HLA matching	33/35 (94.3)	0	33/33 (100)	17/33 (51.5)	9/17 (52.9)	5/9 (55.5)	N/A	2/3 (66.6)	2/2 (100)	2/2 (100)	NGS-SNP combined with appropriate WGA technologies could achieve at least quadruple purposes testing in one PGT cycle.	[41]
α or β thalassemia (217)	--^SEA^	• MDA REPLI-g NGS • PGT-M/PGT-A	94.71	4.26	217/217 (100)	160/ 217 (73.7)	112/ 160 (70)	N/A	32/53 (60.1)	23/53 (43.4)	N/A	N/A	PGT-M could perform without probands and parental pedigrees and high success rate with 100% conclusive results.	[42]
α thalassemia (282)	① α Thal ② α Thal with HLA	• MDA • BlueGnome 24sure BAC array (Illumina-NGS) • PGT-M/ PGT-A	① 254 /259 (98) ② 23/23 (100)	N/A	① 254/254 (100) ② 23/23 (100)	① 146/254 (57.5) ② 6/23 (26.09)	① 231/259 (89.19) ② 3/23 (13)	② 23/23 (100)	① 19/33 (57.3) ② 1/1 (100)	① 17/32 (53.1) ② 1/1 (100)	N/A	N/A	NGS technologies could achieve at multiple purposes testing in one PGT cycle with high conclusive result.	[10]
β thalassemia /Hb E disease (106)	c.126_129 delCTTT c.52A>T c.316–197 C>T c.2T>G c.92+5 G>C	• MDA • NGS • PGT-M/PGT-A	100/ 106 (94.3)	3.89	96/100 (96)	53/100 (53)	100/ 100 (100)	N/A	9/15 (60)	8/15 (53.3)	8/8 (100)	8/8 (100)	NGS is able to perform PGT-A and PGT-M simultaneously and resulted in high accuracy.	[61]
β thalassemia (21)	C92 +6 T→C C118 C→ T C93–21 G→A, C316–106 (C→G) C95 +5 G→C	• MDA • NGS with SNP • PGT-M	21/21 (100)	1/21 (4.8)	20/21 (95.2)	10/21 (47.61)	N/A	N/A	N/A	N/A	N/A	N/A	NGS could provide a rapid, streamlined and potentially cost-effective solution.	[62]
α thalassemia (10, 2)	--^SEA^	• MDA (PicoPLEX Single Cell WGA kit) • HLRS • PGT-M	10/10 (100)	0	10/10 (100)	9/10 (90)	N/A	N/A	2/2 (100)	1/2 (50) 1st TM abortion = 1	1/1 (100)	N/A	HLRS was easy to perform, rich SNPs, no need for familial samples, and high conclusive results.	[53]
β thalassemia (32, 3)	βIVS-II-654 β-90 CD 43	• MDA • NGS • Long read sequencing on SMRT platform (not required probands for haplotype linkage analysis)-NGS • PGT-M	N/A	10/32 (31.2) range 10, 25, 50% in each family	N/A	Haplotype linkage analysis 24/33 (72.7) Correlate with NGS based	N/A	N/A	N/A	N/A	N/A	N/A	Long-read sequencing was the potential tool for preimplantation haplotype linkage analysis for whom cannot receive conventional NGS-SNP.	[45]
β thalassemia (10)	C92 +6 T→C C58 del C	• MDA • NGS • PGT-M with HLA	Allele 84/90 (93.3)	N/A	84/84 (100)	EBs 6/10 (60)	N/A	N/A	N/A	N/A	N/A	N/A	The feasibility of a NGS of preimplantation HLA sequencing was investigated via combining the state-of-the-art techniques used in single-cell whole genome amplification, PGD, and high-resolution HLA typing.	[47]

Abbreviation: ADO, allele drop-out; CGH, comparative genomic hybridization; CP, clinical pregnancy; DGGE, denaturing gradient gel electrophoresis; EB, embryo; ET, embryo transfer; HLRS, haplotyping by linked read sequencing; LB, livebirth; MALBAC, multiple annealing and looping-based amplification cycles; MDA, multiple displacement amplification; *n*, number; NGS, next-generation sequencing; N/A, not applicable; PCR, polymerase chain reaction; PGD, preimplantation genetic diagnosis; PGT, preimplantation genetic testing; PGT-A, preimplantation genetic testing for aneuploidy; PGT-M, preimplantation genetic testing for monogenic disorder; SNP, single nucleotide polymorphism; STR, short tandem repeat; WGA, whole genome amplification.

**Table 4:** Noninvasive preimplantation genetic testing (niPGT-M).

**Disease**	**Sample** **(** ***n*** **)** **① SCM** **②BF+ SCM**	**PGT**				**Method** **Collected Sample**	**WGA Techniques**	**PCR Techniques**	**Outcomes**			**Interpretation**	**Ref.**
		**PGT-A**	**PGT-M**	**PGT-SR**	**PGT-P**				**Amplification Rate (%)**	**ADO Rate (%)**	**Concordance Rate (%)**		
													
α thalassemia	① (202) ∇ compared with biopsied blastomere (413)		/			① collected SCM after moving the blastocyst ∇ biopsied blastomere or cels of embryo day 3	MDA	Quantitative PCR	DNA conc.	① 12	Diagnosis efficiency	The diagnosis efficiency of SCM is significantly increased compared with biopsy based. The optimaltime for medium-based α-thalassemia^-SEA^ detection was Day 5 (D5) following IVF from higher DNA concentration and higher diagnosis efficiency.	[59]
									D4: 14.24, D5 48.78, D6 54.35		D4: 19.67, D5 90.16, D6 88.46		
											∇ 82.1		
β thalassemia	① (33) ② (26)		/			① collected SCM after moving the blastocyst to another drop ②using laser hits zona pellucida to release BF into SCM	MALBAC	NGS	DNA conc. ① 41.5 ± 31.7 ② 147.6 ± 69.8		① 45.5% ② 100%	Detection rate with niPGT using ② was higher than ①. Fragmentation is associated with DNA concentration in ②	[56]

Abbreviation: ADO, allele drop-out; BF, blastocoel fluid; MALBAC, multiple annealing and looping-based amplification cycles; MDA, multiple displacement amplification; *n*, number; NGS, next-generation sequencing; PCR, polymerase chain reaction; PGT, preimplantation genetic testing; PGT-A, preimplantation genetic testing for aneuploidy; PGT-M, preimplantation genetic testing for monogenic disorder; PGT-SR, preimplantation genetic testing for structural rearrangement; PGT-P, preimplantation genetic testing for polygenic disorders; SCM, spent culture medium; WGA, whole genome amplification; /, applicable

## 6. Conclusions

**Figure 1:**
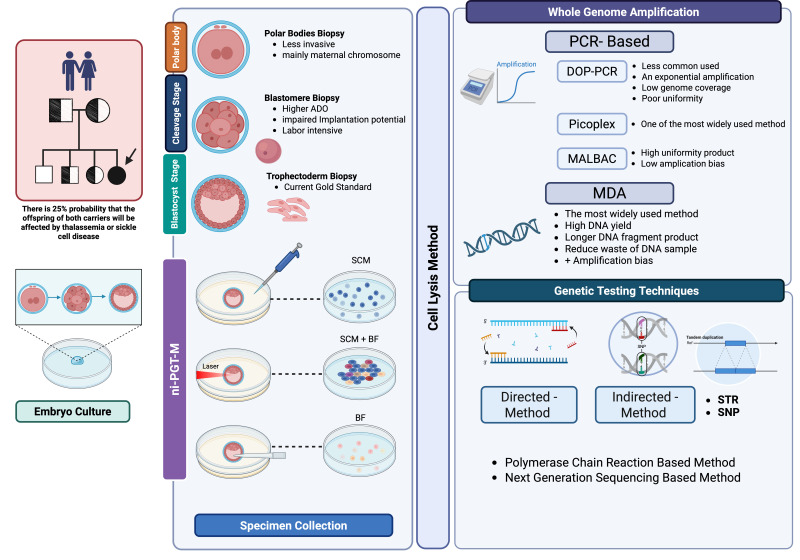
Overview of preimplantation genetic testing for monogenic disorders (PGT-M). Abbreviations: ADO, allele drop-out; BF, blastocoel fluid; DOP-PCR, degenerate oligonucleotide primed polymerase chain reaction; MDA, multiple displacement amplification; MALBAC, multiple annealing and looping-based amplification cycles; SNP, single nucleotide polymorphisms; STR, short tandem repeats. Created in BioRender. Chantrasiri, R. (2025) https://BioRender.com/ojh4ogm.

PGT-M is a rapid, accurate and valuable clinical tool for identifying genetic disorders prior to pregnancy, thereby mitigating the need for selective pregnancy termination and associated ethical dilemmas. Technological advancements in PGT aim to minimize technical errors, such as amplification failure and ADO, thereby enhancing diagnostic accuracy. Trophectoderm biopsy is currently recognized as the gold standard for PGT sampling. This technique, performed at the blastocyst stage, is associated with a lower incidence of ADO and a higher diagnostic reliability compared to the earlier methodology of cleavage-stage blastomere biopsy. Based on our review, MDA is the amplification technique of choice for optimal outcomes, with NGS serving as the primary genetic testing methodology. It is important to note, however, that each of these techniques is associated with distinct advantages and disadvantages. Therefore, clinicians must carefully select the most appropriate technique based on the specific clinical context. While TE biopsy is currently considered the gold standard for sample collection, it is not without drawbacks, including a minimal potential for harm to embryos and the risk of compromising implantation potential, particularly when the procedure is not performed by a highly experienced embryologist. In contrast, minimally invasive and non-invasive PGT-M approaches are emerging as novel techniques that allow for genetic analysis without embryo destruction. However, further research is needed to fully validate niPGT-M methods for routine clinical application (see Figure 1).

## Data Availability

No new data were created or analyzed in this study.

## Abbreviations

The following abbreviations are used in this manuscript:

ADO	allele drop-out
CP	clinical pregnancy
EB	embryo
ET	embryo transfer
LB	livebirth
MALBAC	multiple annealing and looping-based amplification cycles
MDA	multiple displacement amplification
NGS	next-generation sequencing
PCR	polymerase chain reaction
PGD	preimplantation genetic diagnosis
PGT	preimplantation genetic testing
PGT-A	preimplantation genetic testing for aneuploidy
PGT-M	preimplantation genetic testing for monogenic disorder
SNP	single nucleotide polymorphism
STR	short tandem repeat
WGA	whole genome amplification

## References

[1] Mamas T., Kakourou G., Vrettou C., Traeger-Synodinos J. (2022). Hemoglobinopathies and preimplantation diagnostics. Int. J. Lab. Hematol..

[2] Olivieri N.F., Pakbaz Z., Vichinsky E. (2010). HbE/β-thalassemia: Basis of marked clinical diversity. Hematol. Oncol. Clin. N. Am..

[3] Orkin S.H., Kazazian H.H., Antonarakis S.E., Ostrer H., Goff S.C., Sexton J.P. (1982). Abnormal RNA processing due to the exon mutation of beta E-globin gene. Nature.

[4] Somboonchai P., Charoenkwan P., Piyamongkol S., Lattiwongsakorn W., Pantasri T., Piyamongkol W. (2024). Development of pre-implantation genetic testing protocol for monogenic disorders (PGT-M) of Hb H disease. BMC Genom..

[5] Tuo Y., Li Y., Li Y., Ma J., Yang X., Wu S., Jin J., He Z. (2024). Global, regional, and national burden of thalassemia, 1990–2021: A systematic analysis for the global burden of disease study 2021. eClinicalMedicine.

[6] Piyamongkol S., Mongkolchaipak S., Charoenkwan P., Sirapat R., Suriya W., Pantasri T., Tongsong T., Piyamongkol W. (2022). The successful strategy of comprehensive pre-implantation genetic testing for beta-thalassaemia-haemoglobin E disease and chromosome balance using karyomapping. J. Obstet. Gynaecol..

[7] Modell B., Darlison M. (2008). Global epidemiology of haemoglobin disorders and derived service indicators. Bull. World Health Organ..

[8] Fiorentino F., Biricik A., Karadayi H., Berkil H., Karlikaya G., Sertyel S., Podini D., Baldi M., Magli M.C., Gianaroli L. (2004). Development and clinical application of a strategy for preimplantation genetic diagnosis of single gene disorders combined with HLA matching. Mol. Hum. Reprod..

[9] Group E.P.-M.W., Carvalho F., Moutou C., Dimitriadou E., Dreesen J., Gimenez C., Goossens V., Kakourou G., Vermeulen N., Zuccarello D. (2020). ESHRE PGT Consortium good practice recommendations for the detection of monogenic disorders. Hum. Reprod. Open.

[10] Tiewsiri K., Manipalviratn S., Sutheesophon W., Vanichsetakul P., Thaijaroen P., Ketcharoon P., Bradley C.K., McArthur S.J., Krutsawad W., Marshall J.T.A. (2020). The First Asian, Single-Center Experience of Blastocyst Preimplantation Genetic Diagnosis with HLA Matching in Thailand for the Prevention of Thalassemia and Subsequent Curative Hematopoietic Stem Cell Transplantation of Twelve Affected Siblings. Biomed. Res. Int..

[11] Shestak A.G., Bukaeva A.A., Saber S., Zaklyazminskaya E.V. (2021). Allelic Dropout Is a Common Phenomenon That Reduces the Diagnostic Yield of PCR-Based Sequencing of Targeted Gene Panels. Front. Genet..

[12] Chen K., Hu Z., Lian Y., Han Y., Zhou X., Li Y., Xiang L., Jiang W., Li M., Zeng P. (2025). The diagnostic accuracy of preimplantation genetic testing (PGT) in assessing the genetic status of embryos: A systematic review and meta-analysis. Reprod. Biol. Endocrinol..

[13] Lan Y., Zhou H., He S., Shu J., Liang L., Wei H., Luo J., Wang C., Zhao X., Qiu Q. (2023). Appropriate whole genome amplification and pathogenic loci detection can improve the accuracy of preimplantation genetic diagnosis for deletional alpha-thalassemia. Front. Endocrinol..

[14] Piyamongkol W., Vutyavanich T., Sanguansermsri T. (2012). Preimplantation genetic diagnosis of alpha-thalassemia-SEA using novel multiplex fluorescent PCR. J. Assist. Reprod. Genet..

[15] Yang S., Xu B., Zhuang Y., Zhang Q., Li J., Fu X. (2024). Current research status and clinical applications of noninvasive preimplantation genetic testing: A review. Medicine.

[16] Wang J., Lu B.M., Li R., Guo J., Xu Y., Pan J.F., Zeng Y.H., Zhou C.Q., Xu Y.W. (2019). Karyomapping in preimplantation genetic testing for beta-thalassemia combined with HLA matching: A systematic summary. J. Assist. Reprod. Genet..

[17] Chen M., Tan A.S., Cheah F.S., Saw E.E., Chong S.S. (2015). Identification of novel microsatellite markers <1 Mb from the HBB gene and development of a single-tube pentadecaplex PCR panel of highly polymorphic markers for preimplantation genetic diagnosis of beta-thalassemia. Electrophoresis.

[18] Nienhuis A.W., Nathan D.G. (2012). Pathophysiology and Clinical Manifestations of the β-Thalassemias. Cold Spring Harb. Perspect. Med..

[19] Elendu C., Amaechi D.C., Alakwe-Ojimba C.E., Elendu T.C., Elendu R.C., Ayabazu C.P., Aina T.O., Aborisade O., Adenikinju J.S. (2023). Understanding Sickle cell disease: Causes, symptoms, and treatment options. Medicine.

[20] Handyside A.H., Kontogianni E.H., Hardy K., Winston R.M.L. (1990). Pregnancies from biopsied human preimplantation embryos sexed by Y-specific DNA amplification. Nature.

[21] Verlinsky Y., Ginsberg N., Lifchez A., Valle J., Moise J., Strom C.M. (1990). Analysis of the first polar body: Preconception genetic diagnosis. Hum. Reprod..

[22] Dokras A., Sargent I.L., Ross C., Gardner R.L., Barlow D.H. (1990). Trophectoderm biopsy in human blastocysts. Hum. Reprod..

[23] Takeuchi K. (2021). Pre-implantation genetic testing: Past, present, future. Reprod. Med. Biol..

[24] Cimadomo D., Rienzi L., Capalbo A., Rubio C., Innocenti F., Garcia-Pascual C.M., Ubaldi F.M., Handyside A. (2020). The dawn of the future: 30 years from the first biopsy of a human embryo. The detailed history of an ongoing revolution. Hum. Reprod. Update.

[25] Marom Haham L., Aizer A., Arad A., Haas J., Lebovitz O., Zilberberg E., Nahum R., Orvieto R. (2025). The outcomes of blastocyst versus cleavage stage embryo biopsy for preimplantation genetic testing for monogenic diseases. Front. Endocrinol..

[26] Scott R.T., Upham K.M., Forman E.J., Zhao T., Treff N.R. (2013). Cleavage-stage biopsy significantly impairs human embryonic implantation potential while blastocyst biopsy does not: A randomized and paired clinical trial. Fertil. Steril..

[27] Practice Committee and Genetic Counseling Professional Group of the American Society for Reproductive Medicine (2023). Indications and management of preimplantation genetic testing for monogenic conditions: A committee opinion. Fertil. Steril..

[28] Petch S., Crosby D. (2024). Updates in preimplantation genetic testing (PGT). Best Pract. Res. Clin. Obstet. Gynaecol..

[29] Wang X., Liu Y., Liu H., Pan W., Ren J., Zheng X., Tan Y., Chen Z., Deng Y., He N. (2020). Recent advances and application of whole genome amplification in molecular diagnosis and medicine. MedComm.

[30] Bonnette M.D., Pavlova V.R., Rodier D.N., Thompson L.P., Boone E.L., Brown K.L., Meyer K.M., Trevino M.B., Champagne J.R., Cruz T.D. (2009). dcDegenerate oligonucleotide primed-PCR for multilocus, genome-wide analysis from limited quantities of DNA. Diagn. Mol. Pathol..

[31] Kwok P.Y. (2002). Making ‘random amplification’ predictable in whole genome analysis. Trends Biotechnol..

[32] Mai M., Hoyer J.D., McClure R.F. (2004). Use of multiple displacement amplification to amplify genomic DNA before sequencing of the alpha and beta haemoglobin genes. J. Clin. Pathol..

[33] Dadzie F.A., Beaudry M.S., Deyanov A., Slanis H., Duong M.Q., Turner R., Khan A., Arias C.A., Kissinger J.C., Glenn T.C. (2024). Evaluating the Benefits and Limits of Multiple Displacement Amplification with Whole-Genome Oxford Nanopore Sequencing. bioRxiv.

[34] Chapman A.R., He Z., Lu S., Yong J., Tan L., Tang F., Xie X.S. (2015). Single cell transcriptome amplification with MALBAC. PLoS ONE.

[35] Khosravi S., Salehi M., Ramezanzadeh M., Mirzaei H., Salehi R. (2016). Novel Multiplex Fluorescent PCR-Based Method for HLA Typing and Preimplantational Genetic Diagnosis of beta-Thalassemia. Arch. Med. Res..

[36] Kakourou G., Destouni A., Vrettou C., Traeger-Synodinos J., Kanavakis E. (2014). A generic, flexible protocol for preimplantation human leukocyte antigen typing alone or in combination with a monogenic disease, for rapid case work-up and application. Hemoglobin.

[37] Zachaki S., Vrettou C., Destouni A., Kokkali G., Traeger-Synodinos J., Kanavakis E. (2011). Novel and known microsatellite markers within the beta-globin cluster to support robust preimplantation genetic diagnosis of beta-thalassemia and sickle cell syndromes. Hemoglobin.

[38] Kanavakis E., Vrettou C., Palmer G., Tzetis M., Mastrominas M., Traeger-Synodinos J. (1999). Preimplantation genetic diagnosis in 10 couples at risk for transmitting β-thalassaemia major: Clinical experience including the initiation of six singleton pregnancies. Prenat. Diagn..

[39] Fu Y., Shen X., Chen D., Wang Z., Zhou C. (2019). Multiple displacement amplification as the first step can increase the diagnostic efficiency of preimplantation genetic testing for monogenic disease for beta-thalassemia. J. Obstet. Gynaecol. Res..

[40] Farashi S., Harteveld C.L. (2018). Molecular basis of α-thalassemia. Blood Cells Mol. Dis..

[41] Chen D., Shen X., Xu Y., Ding C., Ye Q., Zhong Y., Xu Y., Zhou C. (2021). Successful four-factor preimplantation genetic testing: Alpha- and beta-thalassemia, human leukocyte antigen typing, and aneuploidy screening. Syst. Biol. Reprod. Med..

[42] Ou Z., Deng Y., Liang Y., Chen Z., Sun L. (2022). Using affected embryos to establish linkage phase in preimplantation genetic testing for thalassemia. Reprod. Biol. Endocrinol..

[43] Kuliev A., Pakhalchuk T., Verlinsky O., Rechitsky S. (2011). Preimplantation genetic diagnosis for hemoglobinopathies. Hemoglobin.

[44] Ha Vuong V.V., Nguyen P.-D., Thi N.N., Le Thi P., Minh Nguyet D.T., Nguyen M.H., Tran H.A., Dang-Tran N.-M., Bui T.-H., Tran T.H. (2024). Application of short tandem repeats (STRs) in the preimplantation genetic diagnosis (PGD) of α-thalassemia. Taiwan. J. Obstet. Gynecol..

[45] Wu H., Chen D., Zhao Q., Shen X., Liao Y., Li P., Chiu P.C.N., Zhou C. (2022). Long-read sequencing on the SMRT platform enables efficient haplotype linkage analysis in preimplantation genetic testing for beta-thalassemia. J. Assist. Reprod. Genet..

[46] Natesan S.A., Bladon A.J., Coskun S., Qubbaj W., Prates R., Munne S., Coonen E., Dreesen J.C., Stevens S.J., Paulussen A.D. (2014). Genome-wide karyomapping accurately identifies the inheritance of single-gene defects in human preimplantation embryos in vitro. Genet. Med..

[47] Rafati M., Akhondi M.M., Sadeghi M.R., Tara S.Z., Ghaffari S.R. (2018). Preimplantation High-Resolution HLA Sequencing Using Next Generation Sequencing. Biol. Blood Marrow Transplant..

[48] Krüger J., Schleinitz D. (2017). Genetic Fingerprinting Using Microsatellite Markers in a Multiplex PCR Reaction: A Compilation of Methodological Approaches from Primer Design to Detection Systems In Methods and Protocals.

[49] Foissac A., Salhi M., Cambon-Thomsen A. (2000). Microsatellites in the HLA region: 1999 update. Tissue Antigens.

[50] Hu T., Chitnis N., Monos D., Dinh A. (2021). Next-generation sequencing technologies: An overview. Hum. Immunol..

[51] Mardis E.R. (2013). Next-generation sequencing platforms. Annu. Rev. Anal. Chem..

[52] Chen D., Shen X., Wu C., Xu Y., Ding C., Zhang G., Xu Y., Zhou C. (2020). Eleven healthy live births: A result of simultaneous preimplantation genetic testing of alpha- and beta-double thalassemia and aneuploidy screening. J. Assist. Reprod. Genet..

[53] Li Q., Mao Y., Li S., Du H., He W., He J., Kong L., Zhang J., Liang B., Liu J. (2020). Haplotyping by linked-read sequencing (HLRS) of the genetic disease carriers for preimplantation genetic testing without a proband or relatives. BMC Med. Genom..

[54] Minasi M.G., Fiorentino F., Ruberti A., Biricik A., Cursio E., Cotroneo E., Varricchio M.T., Surdo M., Spinella F., Greco E. (2017). Genetic diseases and aneuploidies can be detected with a single blastocyst biopsy: A successful clinical approach. Hum. Reprod..

[55] Chen H.F., Chen M., Ho H.N. (2020). An overview of the current and emerging platforms for preimplantation genetic testing for aneuploidies (PGT-A) in in vitro fertilization programs. Taiwan. J. Obstet. Gynecol..

[56] Ou Z., Deng Y., Liang Y., Chen Z., Sun L. (2021). Improved Non-Invasive Preimplantation Genetic Testing for Beta-Thalassemia Using Spent Embryo Culture Medium Containing Blastocoelic Fluid. Front. Endocrinol..

[57] Karami N., Iravani F., Bakhshandeh Bavarsad S., Asadollahi S., Mehdi Hoseini S., Montazeri F., Mehdi Kalantar S. (2023). Comparing the advantages, disadvantages and diagnostic power of different non-invasive pre-implantation genetic testing: A literature review. Int. J. Reprod. Biomed..

[58] Hammond E.R., Shelling A.N., Cree L.M. (2016). Nuclear and mitochondrial DNA in blastocoele fluid and embryo culture medium: Evidence and potential clinical use. Hum. Reprod..

[59] Wu H., Ding C., Shen X., Wang J., Li R., Cai B., Xu Y., Zhong Y., Zhou C. (2015). Medium-based noninvasive preimplantation genetic diagnosis for human α-thalassemias-SEA. Medicine.

[60] Herreros M., Marti L., Diaz N., Tio M.C., Rodriguez-Arnedo A., Guerrero J., Ortiz J.A., Bernabeu A., Bernabeu R., Ten J. (2024). Impact of Group vs Individual Embryo Culture Strategies on Blastocyst and Clinical Outcomes. Reprod. Sci..

[61] Satirapod C., Sukprasert M., Panthan B., Charoenyingwattana A., Chitayanan P., Chantratita W., Choktanasiri W., Trachoo O., Hongeng S. (2019). Clinical utility of combined preimplantation genetic testing methods in couples at risk of passing on beta thalassemia/hemoglobin E disease: A retrospective review from a single center. PLoS ONE.

[62] Kubikova N., Babariya D., Sarasa J., Spath K., Alfarawati S., Wells D. (2018). Clinical application of a protocol based on universal next-generation sequencing for the diagnosis of beta-thalassaemia and sickle cell anaemia in preimplantation embryos. Reprod. Biomed. Online.

